# Isatuximab with pomalidomide-dexamethasone in relapsed/refractory multiple myeloma: post-marketing surveillance in Japan

**DOI:** 10.1007/s12185-024-03800-5

**Published:** 2024-05-29

**Authors:** Nami Tagami, Michihiro Uchiyama, Kenshi Suzuki, Heigoroh Shirai, Takeshi Seto, Satoshi Nishina, Shinsuke Iida

**Affiliations:** 1 Oncology Medical in Specialty Care, Sanofi K.K., Tokyo, Japan; 2 Department of Hematology, Japanese Red Cross Society Suwa Hospital, Suwa, Japan; 3 Myeloma/Amyloidosis Center, Japanese Red Cross Medical Center, Tokyo, Japan; 4 Medical Affairs, Post-Authorization Regulatory Studies, Sanofi K.K., Tokyo, Japan; 5 Department of Hematology and Oncology, Institute of Medical and Pharmaceutical Sciences, Nagoya City University, Kawasaki 1, Mizuno-cho, Mizuno-ku, Nagoya City, Aichi 467-8601 Japan

**Keywords:** Isatuximab, Japan, Real-world, Relapsed or refractory multiple myeloma, Safety

## Abstract

This post-marketing surveillance assessed the safety and effectiveness of isatuximab plus pomalidomide and dexamethasone (Isa-Pd) for relapsed or refractory multiple myeloma (RRMM) during real-world use in Japan. Data from 211 individuals with RRMM treated with Isa-Pd in Japan between October 2020 and October 2021 were collected, with follow-up for up to 12 months after initiation of Isa-Pd or until treatment discontinuation. The incidence of adverse drug reactions (ADRs), ADRs of special interest (infusion reactions, bone marrow suppression, infections, cardiac disorders, other ADRs of Grade ≥ 3), and serious ADRs was assessed. Best overall response and overall response rate (ORR) were determined. In the safety analysis set (n = 120), ADR incidence was 57.5%. Most ADRs were hematologic, and serious ADRs occurred in 28.3%. Bone marrow suppression occurred in 46.7% of participants (19.2% serious), infusion reactions in 18.3% (6.7% serious), infections in 11.7% (8.3% serious), and a serious cardiac disorder in one participant; other Grade ≥ 3 ADRs were reported in 3.3% (1.7% serious). In the effectiveness analysis set (n = 108), the most common best overall response was very good partial response (24.1%), and ORR was 51.9%. These findings support the safety and effectiveness of Isa-Pd for RRMM in real-life settings in Japan.

**Supplementary Information:**

The online version contains supplementary material available at 10.1007/s12185-024-03800-5.

## Introduction

Multiple myeloma (MM) is a common hematologic malignancy, in which there is abnormal proliferation of plasma cells in the bone marrow [1, 2]. These abnormal plasma cells secrete monoclonal immunoglobulin proteins, which accumulate and cause the clinical manifestations of MM (i.e., hypercalcemia, renal insufficiency, anemia, and bone lesions), as well as an increased risk of infection [2]. As a result, MM is associated with considerable morbidity and mortality [3, 4].

In Japan, there were approximately 7600 new cases of MM in 2019, with 4500 MM-related deaths, and the incidence of the disease is expected to increase as the population ages [5].

Despite advances in treatment, including autologous stem cell transplantation, proteasome inhibitors (PIs), and immunomodulatory drugs (IMIDs), MM is an incurable disease, and most affected individuals eventually experience relapse due to the development of drug resistance [6]. Efforts to improve survival in individuals with relapsed and/or refractory multiple myeloma (RRMM) have included the development of monoclonal antibodies that target surface proteins expressed by malignant plasma cells, such as SLAMF7 and CD38 [7].

Isatuximab, an anti-CD38 monoclonal antibody [8], was approved in 2020 in Japan for use in combination with pomalidomide plus dexamethasone (Isa-Pd) in patients with RRMM who have received at least two prior treatments [9]. This approval was based on the results of the ICARIA-MM study [10, 11], a global phase 3 study with progression-free survival (PFS) as the primary endpoint. New isatuximab-containing regimens (isatuximab + carfilzomib + dexamethasone [Isa-Kd], isatuximab monotherapy, and isatuximab + dexamethasone) were subsequently approved in Japan in 2021 [12], supported by the findings of three further studies that also had PFS as the primary endpoint (IKEMA [13], ISLANDs [14], and TED10893 [15], respectively).

The ICARIA-MM study demonstrated significantly prolonged PFS and overall survival (OS; key secondary endpoint) with Isa-Pd versus pomalidomide plus dexamethasone alone in individuals with RRMM [10, 11]. In the study, nine individuals from Japan were included in the Isa-Pd arm [16].

As a result of the small number of Japanese individuals included in the ICARIA-MM study, the Japanese Pharmaceuticals and Medical Devices Agency (PMDA) requested an all-case post-marketing assessment of isatuximab as a condition of approval in Japan [9]. To comply with this requirement, mandatory post-marketing surveillance (PMS) was conducted to collect data on the use of isatuximab in Japanese patients. The aim of this PMS was to investigate the safety and effectiveness of Isa-Pd therapy in individuals with RRMM in Japan who received this treatment under conditions of real-world use.

## Materials and methods

### Study design

This PMS was a multicenter, uncontrolled, non-comparative, observational survey. Data from all individuals with RRMM who were treated with isatuximab in Japan were collected via a central registry, using an electronic data capture (EDC) system and case report forms (CRFs). Paper CRFs were used if an institution was unable to use the EDC system. The information collected from each individual is outlined in Supplementary Table S1.

Participant registration was preferably done before the start of isatuximab treatment, but could occur within 14 days of treatment initiation. Participants were followed for up to 12 months after the start of isatuximab administration or until treatment discontinuation due to (i) disease progression or occurrence of an adverse event (AE) that necessitated discontinuation of isatuximab treatment; (ii) the participant no longer visiting the hospital or being transferred to another hospital during the observation period; or (iii) the investigating physician determining that continued isatuximab treatment was inappropriate. Data were collected from participants as per real-world use, with no schedule of visits specified.

The PMS was performed in compliance with the guidelines for Good Post-marketing Study Practice (GPSP) in Japan.

### Study population and treatment

All individuals treated with isatuximab for RRMM at Japanese medical institutions from October 31, 2020, to October 31, 2021 were registered. This analysis includes data only from those individuals who consented to the publication of their data. Because isatuximab is approved in Japan for use in combination with pomalidomide and dexamethasone [9], all participants in this PMS were receiving Isa-Pd. Isatuximab 10 mg/kg was administered by intravenous infusion. The 28-day treatment cycles consisted of four infusions (on Days 1, 8, 15, and 22) in the first cycle and two infusions (on Days 1 and 15) in subsequent cycles.

### Outcome measures

#### Safety

An AE was considered to be an adverse drug reaction (ADR) if a causal relationship with isatuximab could not be ruled out. AEs were classified using the preferred terms (PT) and system organ classes (SOC) of the Medical Dictionary for Regulatory Activities/Japanese version. Multiple ADRs in the same individual were counted as one instance of an ADR when calculating the total number of individuals who experienced ADRs; multiple occurrences of the same ADR in the same individual were counted as one event. On the recommendation of the PMDA, the incidence of four ADRs of special interest (i.e., infusion reactions, bone marrow suppression, infections, and cardiac disorders) was assessed. The incidence of other ADRs of special interest not considered to be related to infusion reactions, bone marrow suppression, infections or cardiac disorders that were of Grade ≥ 3 (according to Common Terminology Criteria for Adverse Events; version 5.0) was assessed, as was the incidence of serious ADRs. A serious ADR was defined as an ADR that resulted in permanent or significant disability/dysfunction, death or birth defects; was life threatening, required hospitalization or extension of a hospital stay; or was considered to have caused any other medically important condition.

The status of ADRs at the time of this analysis (resolved/resolving/unresolved) was also calculated. For this analysis, all ADRs were assessed, including multiple occurrences of the same ADR in same individual.

#### Effectiveness

The effectiveness of isatuximab was assessed using the International Myeloma Working Group (IMWG) response criteria [17] at the end of the final cycle of treatment. Response was classified as stringent complete response (sCR), complete response (CR), very good partial response (VGPR), or partial response (PR). Best overall response (from the response criteria mentioned above) and the overall response rate (sCR + CR + VGPR + PR) were determined.

### Statistical analysis

A safety analysis population of 100 individuals was planned. This was based on the incidences of infusion reactions, bone marrow suppression, infections, and cardiac disorders in the isatuximab arm of the ICARIA-MM study of 72.4%, 57.9%, 80.9%, and 14.5%, respectively [18]. Assuming that these ADRs would occur with similar incidences in clinical practice, it was estimated that a sample size of 100 would provide, at a probability of ≥ 95%, the detection of 65, 50, 74, and 9 events of infusion reaction, bone marrow suppression, infection, and cardiac disorder, respectively.

Descriptive statistics were used to summarize data (i.e., mean and standard deviation [SD], and median and range [minimum, maximum]).

An analysis of the effect of background factors (sex, race, age, body weight, inpatient/outpatient status, International Staging System [ISS] staging, revised-ISS staging, Eastern Cooperative Oncology Group [ECOG] performance status, history of medical complications [hepatic dysfunction, renal impairment, other], current medical complications [hepatic dysfunction, renal impairment, other], history of non-pharmacological treatment, use of concomitant medication for treatment of conditions other than RRMM, and use of concomitant therapy for RRMM) on the safety and effectiveness of isatuximab treatment was performed using Fisher’s exact test and the Cochran-Armitage test, with a two-side significance level set at 5%. The Cochran-Armitage test was used when the factors under consideration were ordinal data with three or more categories; Fisher's exact test was used otherwise.

This was a single-group exploratory analysis and was not intended to test hypotheses. Missing data were not imputed. Data were analyzed using SAS® software, version 9.4 or later.

## Results

### Study population

The registry was initiated (first participant registered) on October 20, 2020; the completion date (last participant completed) was April 19, 2022.

**Fig. 1:**
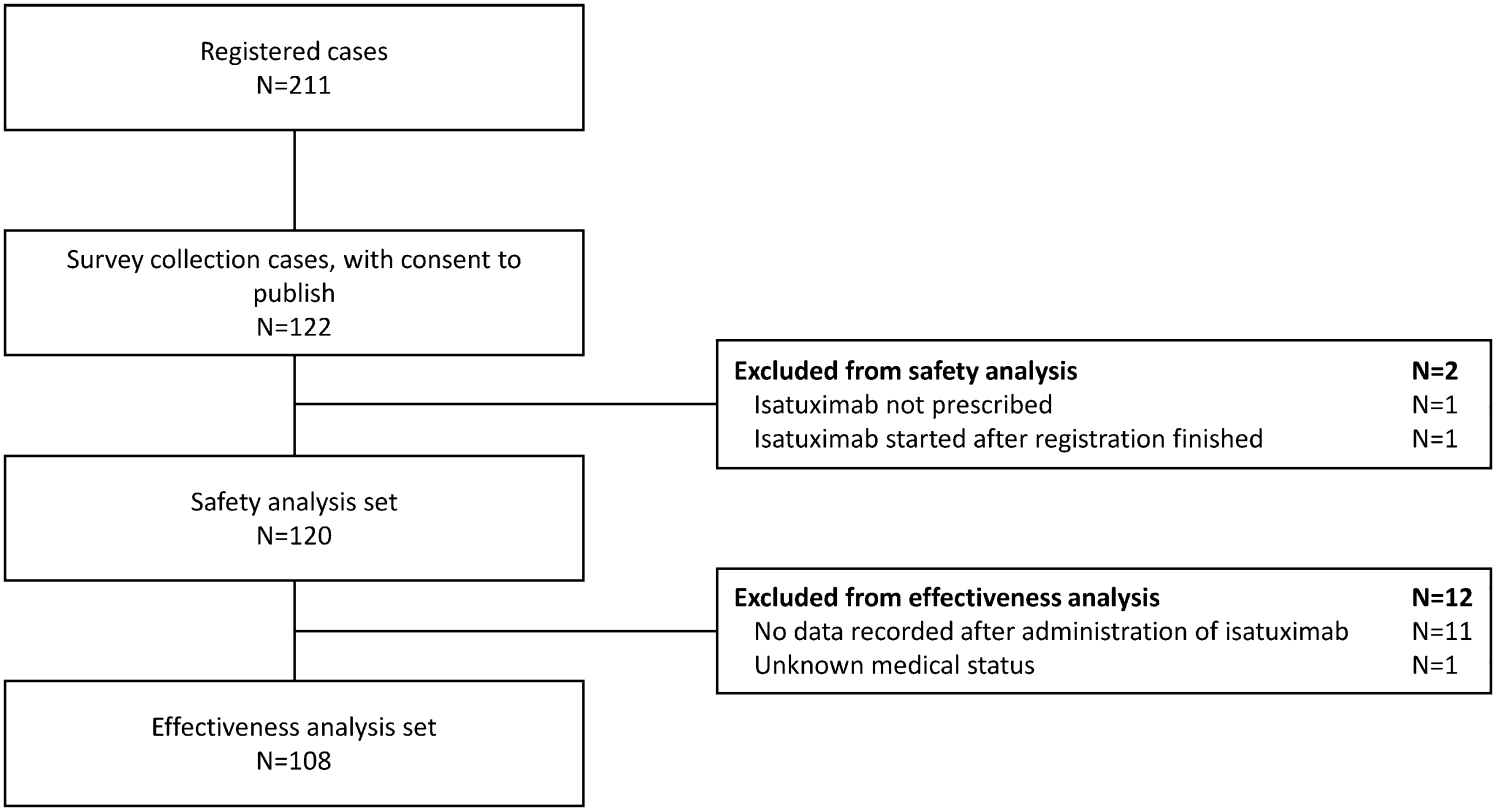
Disposition of the study population

In total, 211 individuals from 122 sites were registered. Of the 122 individuals from whom consent for publication was obtained (including seven individuals whose survey sheets were not collected), 120 were included in the safety analysis (Fig. 1). Twelve individuals were excluded from the effectiveness analysis due to no data recorded after isatuximab administration or unknown medication status; therefore, 108 individuals were included in the effectiveness analysis set.

The majority of participants in the safety analysis set were Japanese (99.2%) and inpatients (90.8%), and 59.2% of participants were male (Table 1). The mean age ± SD was 70.2 ± 9.2 years, and the majority of participants (74.2%) were ≥ 65 years old.

**Table 1:** Baseline demographics and clinical characteristics in the safety analysis and effectiveness analysis sets^a^

	Safety analysis set (N = 120)	Effectiveness analysis set (N = 108)
Sex, n (%)		
Male	71 (59.2)	63 (58.3)
Female^b^	49 (40.8)	45 (41.7)
Race, n (%)		
Asian (Japanese)	119 (99.2)	107 (99.1)
Caucasian	1 (0.8)	1 (0.9)
Age, years		
Mean ± SD	70.2 ± 9.2	
Median (range)	71.0 (46–90)	
Age categories, n (%)		
45–55 years	8 (6.7)	5 (4.6)
55–65 years	23 (19.2)	21 (19.4)
65–75 years	51 (42.5)	46 (42.6)
≥ 75 years	38 (31.7)	36 (33.3)
Treatment location, n (%)		
Inpatient (hospitalized)	109 (90.8)	97 (89.8)
Outpatient	11 (9.2)	11 (10.2)
ISS staging, n (%)^c^		
Stage I	16 (13.3)	14 (13.0)
Stage II	44 (36.7)	42 (38.9)
Stage III	43 (35.8)	36 (33.3)
Unknown/not described	17 (14.2)	16 (14.8)
R-ISS staging, n (%)^c^		
Stage I	10 (8.3)	10 (9.3)
Stage II	47 (39.2)	42 (38.9)
Stage III	30 (25.0)	28 (25.9)
Unknown/not described	33 (27.5)	28 (25.9)
ECOG performance status, n (%)		
0	25 (20.8)	22 (20.4)
1	51 (42.5)	46 (42.6)
2	30 (25.0)	29 (26.9)
3	12 (10.0)	10 (9.3)
4	1 (0.8)	1 (0.9)
Unknown/not stated	1 (0.8)	0
History of medical complications, n (%)		
No	63 (52.5)	58 (53.7)
Yes	57 (47.5)	50 (46.3)
Liver dysfunction	3 (2.5)	2 (1.9)
Renal impairment	5 (4.2)	4 (3.7)
Other	56 (46.7)	49 (45.4)
Current medical complications, n (%)		
No	51 (42.5)	44 (40.7)
Yes	69 (57.5)	64 (59.3)
Liver dysfunction	6 (5.0)	6 (5.6)
Renal impairment	14 (11.7)	13 (12.0)
Other	65 (54.2)	60 (55.6)
Concomitant drug, n (%)		
No	37 (30.8)	32 (29.6)
Yes	83 (69.2)	76 (70.4)
Anti-infective agents	55 (66.3)^d^	
Agents to reduce risk of infusion reaction	75 (90.4)^d^	
Concomitant therapy for RRMM,^e^ n (%)		
No	109 (90.8)	98 (90.7)
Yes	11 (9.2)	10 (9.3)
Previous therapy for MM^f^		
Pl-based	85 (70.8)	
Bortezomib	4 (3.3)	
Bortezomib/dexamethasone	46 (38.3)	
Bortezomib/dexamethasone/panobinostat	6 (5.0)	
Bortezomib/cyclophosphamide/dexamethasone	21 (17.5)	
Carfilzomib	1 (0.8)	
Carfilzomib/dexamethasone	49 (40.8)	
IMiD-based	60 (50.0)	
Thalidomide/dexamethasone	1 (0.8)	
Lenalidomide/dexamethasone	50 (41.7)	
Pomalidomide/dexamethasone	29 (24.2)	
Pl/IMiD-based	68 (56.7)	
Ixazomib/lenalidomide/dexamethasone	34 (28.3)	
Bortezomib/thalidomide/dexamethasone	2 (1.7)	
Bortezomib/lenalidomide/dexamethasone	34 (28.3)	
Bortezomib/pomalidomide/dexamethasone	11 (9.2)	
Carfilzomib/lenalidomide/dexamethasone	23 (19.2)	
Carfilzomib/pomalidomide/dexamethasone	1 (0.8)	
mAb-based	69 (57.5)	
Elotuzumab/lenalidomide/dexamethasone	17 (14.2)	
Elotuzumab/pomalidomide/dexamethasone	27 (22.5)	
Daratumumab/lenalidomide/dexamethasone	34 (28.3)	
Daratumumab/bortezomib/dexamethasone	23 (19.2)	
Daratumumab	2 (1.7)	
Others	79 (65.8)	

*ECOG* Eastern Cooperative Oncology Group; *IMiD* immunomodulatory drug; *ISS* International Staging System; *mAb* monoclonal antibody; *MM* multiple myeloma; *PI* proteasome inhibitor; *R-ISS* revised International Staging System; *RRMM* relapsed and/or refractory multiple myeloma; *SD* standard deviation

^a^All individuals were diagnosed with RRMM and had been previously treated

^b^No females were pregnant

^c^ISS and R-ISS stages were determined just prior to initiation of isatuximab (plus pomalidomide and dexamethasone) therapy or at initial diagnosis

^d^Denominator is the number of patients who received a concomitant drug

^e^Treatments/procedures other than drug therapy received for RRMM (e.g., hematopoietic stem cell transplantation or radiation therapy)

^f^Participants may have received more than one previous therapy for RRMM

All participants had RRMM, with 87 participants having ISS stage II or III disease (36.7% and 35.8%, respectively) and 77 participants having revised-ISS stage II or III disease (39.2% and 25.0%, respectively). ECOG performance status score indicated that disease impact was minimal (score of 0 or 1) in 76 participants (20.8% and 42.5%, respectively). Few participants had current liver dysfunction or renal impairment (5.0% and 11.7%, respectively); 54.2% of participants had other complications.

The mean ± SD duration of isatuximab treatment was 182.5 ± 144.2 days. The mean ± SD isatuximab dose was 10.0 ± 0.2 mg/kg per dose. The mean ± SD duration of treatment and dose of pomalidomide and dexamethasone are provided in Supplementary Table S2.

Of the 120 participants included in the safety analysis set, 32 (26.7%) completed ≥ 12 months of observation, while follow-up ended before this time in 88 (73.3%), most commonly because of primary disease progression (n = 47; 39.2%). AEs (leukopenia, neutropenia, acute myocardial infarction, pneumonia, pulmonary embolism, cytomegalovirus chorioretinitis, hepatic function abnormal, enterocolitis, pneumonia bacterial infection, and fatigue) led to treatment termination in 10 participants. Other reasons for treatment termination are provided in Table 2.

**Table 2:** Status of the safety analysis set

Participants, n (%)	N = 120
Completed ≥ 12 months’ observation	32 (26.7)
Completed < 12 months’ observation	88 (73.3)
Reasons for treatment termination	
Progression of primary disease	47 (39.2)
AE	10 (8.3)
Leukopenia	1 (0.8)
Neutropenia	1 (0.8)
Acute myocardial infarction	1 (0.8)
Pneumonia	1 (0.8)
Pulmonary embolism	1 (0.8)
Cytomegalovirus chorioretinitis	1 (0.8)
Hepatic function abnormal	1 (0.8)
Enterocolitis	1 (0.8)
Pneumonia bacterial infection	1 (0.8)
Fatigue	1 (0.8)
Death	11 (9.2)
No hospital visits	9 (7.5)
Patient convenience/preference^a^	3 (2.5)
Other	8 (6.7)
Status unknown	0

*AE* adverse event

^a^Other than an AE

The demographics and characteristics of the effectiveness analysis set were similar to those of the safety analysis set (Table 1).

### Safety

In the safety analysis set, ADRs were observed in 69 participants (57.5%). The most common ADRs were neutrophil count decreased (n = 31; 25.8%), platelet count decreased (n = 15; 12.5%), anemia (n = 12; 10.0%), neutropenia (n = 9; 7.5%), and white blood cell count decreased (n = 9; 7.5%; Table 3). Serious ADRs were observed in 34 participants (28.3%), with the most common being neutropenia (n = 9; 7.5%), neutrophil count decreased (n = 6; 5.0%), febrile neutropenia (n = 6; 5.0%), pneumonia (n = 4; 3.3%), and white blood cell count decreased (n = 4; 3.3%; Table 3).

**Table 3:** Incidence of adverse drug reaction events in the safety analysis set (N = 120)

ADRs, n (%) System organ class Preferred term	All^a^	Serious^a^	Grade ≥ 3^a,b^	
			Grade 3	Grade 4
Total	69 (57.5)	34 (28.3)	41 (34.2)	31 (25.8)
Infections and infestations	13 (10.8)	10 (8.3)	5 (4.2)	2 (1.7)
Bronchitis	1 (0.8)	0	0	0
Bronchopulmonary aspergillosis	1 (0.8)	1 (0.8)	0	0
Infection	1 (0.8)	0	0	0
Listeriosis	1 (0.8)	1 (0.8)	1 (0.8)	0
Pneumonia	4 (3.3)	4 (3.3)	2 (1.7)	0
Sepsis	2 (1.7)	2 (1.7)	0	2 (1.7)
Septic shock	1 (0.8)	1 (0.8)	1 (0.8)	0
Cytomegalovirus chorioretinitis	1 (0.8)	1 (0.8)	1 (0.8)	0
Bacterial pneumonia	1 (0.8)	1 (0.8)	1 (0.8)	0
Tick dermatitis	1 (0.8)	0	0	0
Blood and lymphatic system disorders	25 (20.8)	18 (15.0)	13 (10.8)	12 (10.0)
Anemia	12 (10.0)	0	7 (5.8)	1 (0.8)
Aplasia pure red cell	1 (0.8)	1 (0.8)	0	1 (0.8)
Febrile neutropenia	6 (5.0)	6 (5.0)	6 (5.0)	0
Leukopenia	3 (2.5)	3 (2.5)	3 (2.5)	0
Bone marrow suppression	1 (0.8)	1 (0.8)	0	1 (0.8)
Neutropenia	9 (7.5)	9 (7.5)	1 (0.8)	8 (6.7)
Pancytopenia	1 (0.8)	1 (0.8)	0	1 (0.8)
Metabolic and nutritional disorders	1 (0.8)	1 (0.8)	0	1 (0.8)
Hypercalcemia	1 (0.8)	1 (0.8)	0	1 (0.8)
Mental disorder	1 (0.8)	1 (0.8)	unknown	unknown
Delirium	1 (0.8)	1 (0.8)	unknown	unknown
Cardiac disorder	1 (0.8)	1 (0.8)	0	0
Heart failure	1 (0.8)	1 (0.8)	0	0
Vascular disorder	4 (3.3)	0	0	0
Hypertension	2 (1.7)	0	0	0
Hypotension	2 (1.7)	0	0	0
Respiratory, thoracic and mediastinal disorders	10 (8.3)	2 (1.7)	2 (1.7)	0
Difficulty breathing	3 (2.5)	0	0	0
Hypoxia	2 (1.7)	2 (1.7)	2 (1.7)	0
Nasal congestion	1 (0.8)	0	0	0
Rhinorrhea	1 (0.8)	0	0	0
Wheezing	2 (1.7)	0	1 (0.8)	0
Inflammation of the upper respiratory tract	1 (0.8)	0	0	0
Oropharyngeal discomfort	3 (2.5)	0	0	0
Gastrointestinal disorders	3 (2.5)	1 (0.8)	1 (0.8)	1 (0.8)
Enterocolitis	1 (0.8)	1 (0.8)	0	0
Nausea	1 (0.8)	0	1 (0.8)	0
Vomiting	1 (0.8)	0	0	1 (0.8)
Hepatobiliary disorders	1 (0.8)	1 (0.8)	0	1 (0.8)
Hepatic function abnormal	1 (0.8)	1 (0.8)	0	1 (0.8)
General and systemic disorders and administration site conditions	7 (5.8)	1 (0.8)	4 (3.3)	1 (0.8)
Chest discomfort	1 (0.8)	0	1 (0.8)	0
Chills	2 (1.7)	0	1 (0.8)	0
Malaise	1 (0.8)	0	1 (0.8)	0
Fever	2 (1.7)	0	1 (0.8)	1 (0.8)
Disease progression	2 (1.7)	1 (0.8)	1 (0.8)	0
Investigations	41 (34.2)	8 (6.7)	21 (17.5)	19 (15.8)
Heart rate increased	1 (0.8)	0	0	0
Neutrophil count decreased	31 (25.8)	6 (5.0)	15 (12.5)	14 (11.7)
Platelet count decreased	15 (12.5)	2 (1.7)	6 (5.0)	5 (4.2)
White blood cell count decreased	9 (7.5)	4 (3.3)	2 (1.7)	4 (3.3)
Injury, poisoning and procedural complications	8 (6.7)	0	0	0
Infusion-related reactions^c^	8 (6.7)	0	0	0

*ADR* adverse drug reaction

^a^Each participant could have experienced more than one ADR, serious ADR and/or ADR event of each severity grade

^b^All ADRs were of Grade 3–4; no Grade 5 ADRs were reported

^c^Localized reactions associated with the infusion procedure

Of the 69 participants who had experienced an ADR, 58 participants (48.3%) had an ADR that was resolved at last follow-up, 19 participants (15.8%) had resolving ADRs, seven participants (5.8%) had unresolved ADRs, and one participant (0.8%) died (due to disease progression). Three participants (2.5%) were with an unknown outcome associated with the ADR. Of note, some participants experienced more than one ADR, each with a potentially different outcome.

#### ADRs of special interest

In total, at least one infusion reaction ADR occurred in 22 participants (18.3%), bone marrow suppression ADRs occurred in 56 participants (46.7%), infectious disease ADRs occurred in 14 participants (11.7%), and cardiac disorder occurred in one participant (0.8%; Table 4). Eight, 23, 10, and one of these ADRs, respectively, were considered serious.

**Table 4:** Number of participants in the safety analysis set (N = 120) with adverse drug reactions of special interest

ADRs of special interest, n (%)	All^a^	Serious^a^	Grade ≥ 3^a,b^	
			Grade 3	Grade 4
Infusion reaction^c^	22 (18.3)	8 (6.7)	6 (5.0)	11 (9.2)
Bone marrow suppression^d^	56 (46.7)	23 (19.2)	34 (28.3)	29 (24.2)
Infectious disease^e^	14 (11.7)	10 (8.3)	6 (5.0)	2 (1.7)
Cardiac disorders^f^	1 (0.8)	1 (0.8)	0	0
Other Grade ≥ 3 ADRs of special interest^g^	4 (3.3)	2 (1.7)	–	–

*ADR* adverse drug reactions; *MedDRA PT(s)* Medical Dictionary for Regulatory Activities preferred term(s)

^a^Each participant could have experienced more than one ADR, and more than one serious ADR and ADR of each severity grade

^b^All ADRs were of Grade 3–4; no Grade 5 ADRs were reported

^c^Included the following MedDRA PTs: anemia, bronchitis, difficulty breathing, chest discomfort, chills, enterocolitis, fever, heart failure, hypertension, hypotension, hypoxia, increased heart rate, infusion-related reaction, nasal congestion, nausea, neutropenia, neutrophil count decreased, oropharyngeal discomfort, pancytopenia, platelet count decreased, pneumonia, pure red cell aplasia, rhinorrhea, vomiting, wheezing

^d^Included the following MedDRA PTs: anemia, febrile neutropenia, leukopenia, myelosuppression, neutropenia, neutrophil count decreased, pancytopenia, platelet count decreased, pure red cell aplasia, white blood cell count decreased

^e^Included the following MedDRA PTs: bacterial pneumonia, bronchitis, bronchopulmonary aspergillosis, cytomegalovirus chorioretinitis, fever, infection, listeriosis, pneumonia, sepsis, septic shock, tick dermatitis

^f^Included the following MedDRA PTs: heart failure

^g^Included the following MedDRA PTs: hepatic function abnormal, disease progression, hypercalcemia, malaise

Of the 22 participants who experienced an infusion reaction ADR, the ADR had resolved in 22 participants and was resolving in seven participants at last follow-up (some participants experienced more than one infusion reaction ADR). Of the 56 participants who developed a bone marrow suppression ADR, the ADR had resolved in 37 participants and was resolving in 18 participants at last follow-up; five participants had unresolved bone marrow suppression and three participants had an unknown outcome (some participants experienced more than one bone marrow suppression ADR). In 11 of the 14 participants who developed an infection, the ADR resolved; it was resolving in one participant and two had not recovered from the infection at last follow-up. One participant experienced a cardiac disorder ADR (i.e., heart failure), which was considered to be serious; this participant had recovered from the ADR at last follow-up.

Other Grade ≥ 3 ADRs of special interest were reported in four participants (3.3%; Table 4). In two of these participants (1.7%), the ADRs were considered serious (hypercalcemia and hepatic function abnormal). In three of the participants, the ADRs resolved; one participant (with disease progression) did not recover.

In total, 31 participants (25.8%) in the safety analysis set were aged < 65 years and 89 (74.2%) were aged ≥ 65 years. ADRs were reported in 16 (51.6%) and 53 (59.6%) of these participants, respectively. In participants aged < 65 years and ≥ 65 years, infusion reactions were reported in 12.9% (n = 4) and 20.2% (n = 18), respectively; bone marrow suppression in 41.9% (n = 13) and 48.3% (n = 43), respectively; infections in 16.1% (n = 5) and 10.1% (n = 9), respectively; and cardiac disorders in 0% and 1.1% (n = 1), respectively. No participants aged < 65 years had other ADRs of Grade ≥ 3, while 4.5% (n = 4) of those aged ≥ 65 years experienced such an ADR.

#### Effect of background factors on safety

The background factors of participants had no significant effect on overall safety, with the exception of ECOG performance status (p = 0.002), current medical complications (p < 0.001) (specifically renal impairment [p = 0.041], and ‘other’ complications [p < 0.001]), and the use of concomitant drugs (p = 0.046). ADRs were reported more frequently in participants with current medical complications (72.5% [50/69] vs 37.3% [19/51] in those without complications), current renal impairment (85.7% [12/14] vs 53.8% [57/106] in those without) and ‘other’ complications (72.3% [47/65] vs 40.0% [22/55] in those without), and in participants taking concomitant drugs (63.9% [53/83] vs 43.2% [16/37] in those not taking concomitant drugs).

The background factors of participants had no significant effect on the incidence of infusion reactions, infections, or cardiac disorders. However, the incidence of bone marrow suppression was significantly higher in participants with a history of medical complications (57.9% [33/57] vs 36.5% [23/63] in those without a history of complications; p = 0.028), specifically those with a history of ‘other’ complications that were not hepatic dysfunction or renal function impairment (e.g., hypertension, appendicitis, and cataract; 57.1% [32/56] vs 37.5% [24/64]; p = 0.043); and in participants with current medical complications (60.9% [42/69] vs 27.5% [14/51] in those without current complications; p < 0.001), specifically those with current renal impairment (78.6% [11/14] vs 42.5% [45/106]; p = 0.020) and ‘other’ complications (e.g., hypertension, diabetes, and dyslipidemia; 60.0% [39/65] vs 30.9% [17/55]; p = 0.002).

The incidence of other Grade ≥ 3 ADRs was significantly higher in participants taking concomitant therapy for RRMM (18.2% [2/11] vs 1.8% [2/109] in those not taking concomitant therapy for RRMM; p = 0.042).

### Effectiveness

In the effectiveness analysis set, the most common best overall response at the end of the final cycle of treatment was a VGPR (n = 26, 24.1%; Table 5). Five participants (4.6%) achieved a best response of sCR, while four (3.7%) had CR and 21 (19.4%) had PR. The overall response rate was 51.9% (56/108). Minimal response was observed in three participants (2.8%), while stable and progressive disease were seen in 21 (19.4%) and 28 (25.9%) participants, respectively.

**Table 5:** Response to isatuximab-based treatment in the effectiveness analysis set

n (%)	N = 108
Best overall response	
sCR	5 (4.6)
CR	4 (3.7)
VGPR	26 (24.1)
PR	21 (19.4)
Minimal response	3 (2.8)
Stable disease	21 (19.4)
Progressive disease	28 (25.9)
Overall response rate^a^	56 (51.9)

*CR* complete response; *PR* partial response; *sCR* stringent complete response; *VGPR* very good partial response

^a^Overall response = sCR + CR + VGPR + PR

The participant background factors that had a significant effect on the overall response rate were a history of medical complications (p = 0.012), a medical history other than liver dysfunction or renal impairment (p = 0.020), and the use of concomitant drugs (p = 0.011). Specifically, the overall response rate was significantly higher in participants without a history of medical complications (63.8% [37/58] vs 38.0% [19/50] in those with a history of complications) and higher in participants without a history of ‘other’ complications (e.g., hypertension, appendicitis, and cataract; 62.7% [37/59] vs 38.8% [19/49] in those with such a history). The overall response rate was also significantly higher in participants who were not taking concomitant drugs than in those who were taking concomitant drugs (71.9% [23/32] vs 43.4% [33/76]).

When analyzed by previous treatment, the overall response rate was significantly higher in participants who had not previously received daratumumab (67.3% [33/49] vs 39.0% [23/59] in those who had received this treatment; p = 0.004) and in participants who had not previously received daratumumab or pomalidomide (75.0% [18/24] vs 45.2% [38/84] in those who had received this treatment; p = 0.011). Previous treatment with pomalidomide had no significant effect on the overall response rate. Further, the proportion of participants with a response of VGPR or better was numerically higher in participants who had not previously received daratumumab (20/49 [40.8%]), pomalidomide (17/43 [39.5%]), and daratumumab or pomalidomide (10/24 [41.7%]) compared with those who had previously received these treatments (15/59 [25.4%], 18/65 [27.7%], and 25/84 [29.8%], respectively).

## Discussion

This PMS provides evidence of the safety and effectiveness of Isa-Pd for the treatment of individuals with RRMM in Japan.

It is the first analysis of any type to report the safety and effectiveness of Isa-Pd for RRMM in a predominantly Japanese population. At the time of its approval in Japan, data on the efficacy and safety of isatuximab were available from the primary analysis of the phase 3 ICARIA-MM study (n = 307), with a median follow-up of 11.6 months [10]. Subsequently, data from a prespecified 24-month overall survival analysis were also reported [11]. In addition, a subgroup analysis of East Asian study participants (n = 36) was described, which included 13 Japanese individuals, of whom nine received Isa-Pd [16].

In this PMS, ADRs were observed in 57.5% of participants. The most common ADRs were hematologic-related events (i.e., neutrophil count decreased, platelet count decreased, anemia, neutropenia, and white blood cell count decreased), which occurred in > 7% of the participants. The most common non-hematologic ADRs were infusion reactions (6.7%) and pneumonia (3.3%). Serious ADRs occurred in 28.3% of participants, with the most common also being hematologic in nature (i.e., neutropenia, neutrophil count decreased, and febrile neutropenia; each occurring in ≥ 5% of participants). No new safety signals were identified when compared with the treatment-emergent AEs reported in the 24-month analysis of the phase 3 ICARIA-MM study [11].

The most common ADR of special interest was bone marrow suppression, which occurred in 46.7% of participants (19.2% with serious bone marrow suppression). Infusion reactions and infections were also observed in over 10% of participants (18.3% and 11.7%, respectively). The incidence of Grade ≥ 3 infusion reactions (Grade 3, 5.0%; Grade 4, 9.2%) was higher than that reported in the ICARIA-MM study (3%) [11]. Since infusion reactions occurred in 4/9 (44.4%) of the Isa-Pd group in the Japanese cohort of the ICARIA-MM study and in 12/21 (57.1%) of the Asian cohort [16] (i.e., higher than in this PMS [18.3%]), it is unlikely that grade 2 or lower infusion reactions are less common in the Japanese population. Therefore, racial differences are not the likely cause of the lower frequency of infusion reactions in this PMS when compared to previously-published studies. Because data capture was performed under routine medical care, it is reasonable to assume that only serious ADRs requiring infusion reaction supportive care were retrieved from medical records, and that mild infusion reactions were not recorded and underestimated in our study.

However, the incidence of infections in this PMS was lower than that reported in a retrospective study of individuals (n = 107) treated with Isa-Pd in routine care in 24 centers in the UK (23.4%) [19]. Cardiac disorders were very uncommon in the current PMS, reported in only one participant (< 1%).

In the 24-month ICARIA-MM analysis, Isa-Pd was associated with a median PFS of 11.1 months (95% confidence interval [CI] 7.8–13.8) compared with 5.9 months (95% CI 4.5–7.9) with pomalidomide and dexamethasone alone (p < 0.0001) after a median follow-up of 35.3 months [11]. Median OS was 24.6 months (95% CI 20.3–31.3) in the isatuximab group and 17.7 months (95% CI 14.4–26.2) in the control group (p = 0.028) [11]. The subgroup analysis of the ICARIA-MM study [16] reported similar efficacy for Isa-Pd in East Asian individuals to that found in the overall study population [10, 11]. In the current PMS, the observed effectiveness of Isa-Pd (overall response rate of 51.9%) was slightly lower than that reported in the ICARIA-MM study (63%) [11], and in the East Asian (71.4%) and Japanese cohorts (64.4%) [16]. This may have been because of the older age of participants in this PMS (median age 71.0 vs 68 years in the ICARIA-MM study [11], 66.0 years in the overall East Asian cohort, and 67.0 years in the Japanese cohort [16] in those receiving isatuximab). The lower efficacy in this PMS cohort may also be partly due to a large proportion of the participants having had prior exposure to Pd or elotuzumab + Pd and/or an anti-CD38 monoclonal antibody, such as daratumumab, before initiating Isa-Pd. Indeed, the overall response rate in participants of the PMS who had not received prior treatment with daratumumab (67.3%) or daratumumab or pomalidomide (75.0%) was similar to the overall response rate reported in the ICARIA-MM study (in which the vast majority of participants had not previously received daratumumab) [10, 11]. Moreover, the rate of disease progression may have been higher in this PMS than in ICARIA-MM due to the inclusion of individuals with poor ECOG performance status and complications who were treated in routine care; individuals with these characteristics were excluded from the ICARIA-MM study.

Further, the proportion of participants with sCR, CR, VGPR, PR, minimal response, and stable disease in the current PMS (4.6%, 3.7%, 24.1%, 19.4%, 2.8%, and 19.4%, respectively) were similar to those reported in the overall ICARIA-MM study population (< 1%, 9%, 29%, 25%, 6%, and 21%, respectively) [11] and the East Asian cohort (sCR 0%, CR 9.5%, PR 9.5%) [16]. Further, a VGPR or better was observed in a slightly higher proportion of participants in the PMS who had not received prior treatment with daratumumab (40.8%) or daratumumab or pomalidomide (41.7%) than in the ICARIA-MM population (32%) [10]. However, our PMS found a higher rate of progressive disease (25.9%) than in the ICARIA-MM study (5%) [11], and a lower rate of VGPR (24.1%) than in the East Asian and Japanese cohorts of the ICARIA-MM study (52.5% and 44.4%, respectively) [16].

The analysis of the effect of background factors on safety and effectiveness suggests that individuals with a more complex clinical history or with current medical complications who are taking concomitant drugs may be more likely to experience ADRs (particularly bone marrow suppression) and have a less robust treatment response to isatuximab-based treatment. Of note, several chronic comorbidities have previously been associated with an increased risk of chemotherapy-induced bone marrow suppression, including chronic obstructive pulmonary disease, congestive heart failure, human immunodeficiency virus infection, autoimmune disorders, peptic ulcer disease, and thyroid disorders [20]. However, a history of medical complications in itself is likely to be associated with the use of concomitant drugs; thus, these factors may interfere with each other in a multivariate analysis. Further, previous treatment with daratumumab and/or pomalidomide appears to be associated with a less robust response to isatuximab-based treatment; again, this may reflect the impact of a more complex disease history on response.

The current PMS provides real-world data on the use of Isa-Pd to treat RRMM, reflecting routine clinical practice and the experience of individuals with RRMM in Japan, supporting the data obtained from the randomized controlled trial [10, 11]. Furthermore, because all individuals treated with isatuximab at the time of the survey were included in the analysis, these results are likely to be generalizable to future populations who will be treated with this drug in Japan. However, there are some limitations that need to be considered when interpreting these results. Firstly, due to the uncontrolled, observational nature of this PMS, some potential confounders, such as high-risk chromosome abnormalities, were not assessed or accounted for. Secondly, only 120 of the individuals registered provided consent for their data to be published, thus reducing the sample size and the power of the PMS and increasing the likelihood of a type II error. Thirdly, safety data were collected using electronic (or paper) CRFs, which could have led to an underestimation of the number of ADRs in cases where the ADRs were not reported in the CRFs. Finally, while the PMS was designed to assess treatment with isatuximab for up to 12 months, only 32 of the 120 particiapnts received treatment for this time, with 88 participants being treated for < 12 months.

In conclusion, the findings of this PMS indicated the safety and effectiveness of Isa-Pd in the treatment of individuals with RRMM in real-life clinical settings in Japan. These results support the findings of the phase 3 ICARIA-MM study [10, 11], including the subanalysis of individuals from East Asia [16]. No new safety signals were identified in the current PMS, indicating that no additional safety measures are required for the use of isatuximab-based treatment in clinical practice in Japan.

### Supplementary Information

Below is the link to the electronic supplementary material.

Supplementary file1 (DOCX 38 KB)

## Data Availability

Qualified researchers may request access to patient level data and related study documents including the clinical study report, study protocol with any amendments, blank case report form, statistical analysis plan, and dataset specifications. Patient level data will be anonymized and study documents will be redacted to protect the privacy of our trial participants. Further details on Sanofi’s data sharing criteria, eligible studies, and process for requesting access can be found at: https://www.vivli.org/.
