# Supplementary material for: Isatuximab with pomalidomide-dexamethasone in relapsed/refractory multiple myeloma: post-marketing surveillance in Japan
Source: Int J Hematol. 2024 May 29;120(2):217–28. doi: 10.1007/s12185-024-03800-5 (PMC11284182; doi:10.1007/s12185-024-03800-5)
Supplement: Supplementary file 1 — Supplementary file1 (DOCX 38 KB) [file 12185_2024_3800_MOESM1_ESM.docx]

# Supplementary material

## Supplementary Table S1.

Data collected for each study participant

| **Background** |
| --- |
| Identification number; age at start of treatment; date of start of treatment; diagnosis; details of any prior use of isatuximab |
| Demographics: sex; race; weight; inpatient/outpatient status at start of treatment with isatuximab |
| Diagnosis: stage (ISS and R-ISS); date of diagnosis; ECOG performance status at start of treatment |
| Consent for publication of data |
| Past history of medical complications |
| **Non-pharmacological pre-treatment/treatment for MM** |
| Yes/no; treatment; start/end date of treatment |
| **Pharmacological pre-treatment for MM** |
| Yes/no; treatment; start/end date of treatment |
| **Treatment for MM, including isatuximab (per cycle)** |
| Dose; duration of treatment; reason for dose change; suspension after previous treatment; treatment delay; discontinuation; suspension; reason for suspension; resumption and reason |
| **Treatment (other than pomalidomide and dexamethasone) for MM and concomitant use** |
| Name of drug; start and end of treatment; total daily dose (or other dose) |
| **Non-pharmacological combination treatment and treatment for MM** |
| Yes/no; treatment name; treatment start/end date |
| **Effectiveness evaluation for MM** |
| According to the IMWG efficacy criteria |
| **AEs (specified events^a^, or of Grade ≥3 other than specified events^a^)** |
| Incidence and details of AEs: date of onset; name of AE; grade (according to CTCAE, version 5.0); seriousness; treatment; outcome; outcome date/date of outcome confirmation; causality related to isatuximab; possible factors other than isatuximab |
| **Laboratory data, etc. relevant to AEs (specified events^a^, or of Grade ≥3 other than the specified events^a^)** |
| Presence or absence of clinically significant abnormal changes corresponding to AEs, if any; date of collection of relevant laboratory data; measurement results |
| **Survey implementation status** |
| Survey completion/discontinuation; reason; date of death; cause of death; etc. |

^a^Infusion reaction, bone marrow suppression, infections and cardiac disorders

AE(s), adverse event(s); CTCAE, Common Terminology Criteria for Adverse Events; ECOG, Eastern Cooperative Oncology Group; IMWG, International Myeloma Working Group; ISS, International Staging System; MM, multiple myeloma; R-ISS, revised International Staging System

## Supplementary Table S2.

Duration of treatment and dose of pomalidomide and dexamethasone

| **Mean ± SD** | **Pomalidomide (N=118)** | **Dexamethasone (N=118)** |
| --- | --- | --- |
| Duration of treatment (including rest days), days | 178.7 ± 144.1 | 180.4 ± 147.3 |
| Dose per day, mg | 3.1 ± 0.9 | 22.8 ± 12.4 |

SD, standard deviation
